# The effects of synbiotics on indoxyl sulphate level, constipation, and quality of life associated with constipation in chronic haemodialysis patients: a randomized controlled trial

**DOI:** 10.1186/s12882-022-02890-9

**Published:** 2022-07-22

**Authors:** Aida Lydia, Tities Anggraeni Indra, Aulia Rizka, Murdani Abdullah

**Affiliations:** 1 Division of Nephrology and Hypertension, Department of Internal Medicine, Universitas Indonesia/Dr. Cipto Mangunkusumo National Hospital, Jakarta, Indonesia; 2 Department of Internal Medicine, Universitas Indonesia/Dr. Cipto Mangunkusumo National Hospital, Jl. Pangeran Diponegoro No.71, Jakarta, 10430 Indonesia; 3 Division of Geriatrics, Department of Internal Medicine, Universitas Indonesia/Dr. Cipto Mangunkusumo National Hospital, Jakarta, Indonesia; 4 Division of Gastroenterology, Department of Internal Medicine, Universitas Indonesia/Dr. Cipto Mangunkusumo National Hospital, Jakarta, Indonesia

**Keywords:** Synbiotics, Indoxyl sulphate, Constipation, Quality of life, Chronic kidney disease, Haemodialysis

## Abstract

**Background:**

Gut microbiota dysbiosis in patients with chronic kidney disease on haemodialysis (CKD-HD) creates an increase in proteolytic bacteria activity, leading to an increase in the production of uraemic toxins, such as indoxyl sulphate, worsening of constipation symptoms and reducing patients’ quality of life. Improving gut microbiota dysbiosis is expected to improve this condition. This study aimed to evaluate the effect of synbiotics on indoxyl sulphate levels, constipation symptoms, and constipation-related quality of life in haemodialysis patients.

**Methods:**

This was a double-blinded randomized controlled clinical trial with a parallel design involving haemodialysis patients. We included chronic haemodialysis patients with gastrointestinal complaints, difficulty defecating, faeces with hard consistency, or a bowel movement frequency of fewer than three times per week. Patients were randomly divided into two groups (synbiotics (*Lactobacillus acidophilus* and *Bifidobacterium longum* 5x10^9^ CFU) and placebo) for 60 days of oral intervention. All participants, caregivers, and outcome assessors were blinded to group assignment. The primary outcome was a decrease in indoxyl sulphate toxin levels. Meanwhile, improvement in constipation symptoms (measured using the Patient Assessment of Constipation: Symptoms (PAC-SYM) questionnaire) and improvement in constipation-related quality of life (measured using the Patient Assessment of Constipation Quality of Life (PAC-QOL) questionnaire) were assessed as secondary outcomes.

**Results:**

We included 60 patients (30 intervention; median age of 51.23 (13.57) years, 33.3% male; 30 control; median age of 52.33 (11.29) years, 36.7% male). There was no significant difference in terms of pre- and postintervention indoxyl sulphate toxin levels in the synbiotics group compared to the placebo group (*p*=0.438). This study found an improvement in constipation symptoms (*p* = 0.006) and constipation-related quality of life (*p*=0.001) after synbiotic administration.

**Conclusion:**

Two months of synbiotic supplementation did not lower indoxyl sulphate toxin levels. Nevertheless, it had a major effect in improving constipation and quality of life affected by constipation in patients undergoing chronic haemodialysis.

**Trial registration:**

NCT04527640 (date of first registration: 26/08/2020)

**Supplementary Information:**

The online version contains supplementary material available at 10.1186/s12882-022-02890-9.

## Introduction

Dysbiosis is an imbalance in the gut microbiota that causes changes in the number and activities of the microbiota in the gastrointestinal tract. Conditions in chronic kidney disease (CKD) that serve as risk factors for gut microbiota dysbiosis include a low-fibre diet, uraemia, prolonged colonic transit time, disturbance of protein assimilation, use of drugs such as antibiotics, phosphate-binding agents, and iron supplements, as well as other comorbidities [1]. Dysbiosis in CKD is characterized by an increase in the activity of proteolytic bacteria, such as the family *Enterobacteriaceae* (especially *Enterobacter*, *Klebsiella* and *Escherichia*), *Enterococci*, *Clostridium perfringens* and *Pseudomonas,* accompanied by a decrease in the activity of saccharolytic bacteria, such as the family *Bifidobacteriaceae* (especially *Bifidobacterium*, *Lactobacillaceae* and *Prevotellaceae*). This leads to an increase in the production of uraemic toxins, such as indoxyl sulphate (IS), p-cresyl sulphate (p-CS) and indole acetic acid (IAA), as well as a decrease in the production of short-chain fatty acids (SCFAs), such as butyrate and propionate [2].

Indoxyl Sulphate (IS) is a result of tryptophan metabolism by bacteria with the tryptophanase enzyme in the colon. In normal kidneys, this toxin is excreted through the secretion process in the tubules. This toxin is 90% bound to albumin in the circulation and, therefore, cannot be eliminated through the haemodialysis process [3]. As kidney function decreases, the accumulation of IS increases in the body of haemodialysis patients. IS activates nuclear factor-ƙB (NF-ƙB) and the expression of plasminogen activator inhibitor (PAI) type 1 in proximal tubular cells, mediating tubulointerstitial fibrosis, and is strongly associated with the progression of kidney damage. Additionally, IS is associated with aortic calcification, vascular stiffness, and increased oxidative stress in vascular endothelial cells, leading to increased cardiovascular risks in patients with CKD [4].

In addition to being associated with an increase in uraemic toxins, dysbiosis of the gut microbiota is also related to constipation, as it is one of the most frequent gastrointestinal symptoms experienced by haemodialysis patients [5]. Inflammation, increased uraemic toxin, and decreased butyrate acid are suspected to affect intestinal motility [6]. With inadequate treatment, persistent constipation affects patients’ mental and physical quality of life. Zhang *et al*. [7] reported that haemodialysis patients with constipation had lower scores on physical and mental health.

Improvement of dysbiosis by synbiotic administration is expected to lower IS toxin levels, improve constipation symptoms, and enhance constipation-related quality of life. However, currently available studies were conducted with small sample sizes, and not all of them were designed as double-blind randomized clinical trials. The results of these studies vary and cannot provide full evidence of the role of synbiotics in the improvement of gut dysbiosis, decreased uraemic toxins, and gastrointestinal symptoms [8–11].

This study aimed to show the benefit of synbiotic administration in lowering uraemic toxins, specifically IS, and to investigate its benefits on constipation symptoms and the quality of life of haemodialysis patients in Indonesia.

## Methods

### Trial design

This study was a double-blinded randomized controlled clinical trial with a parallel design organized in Dr. Cipto Mangunkusumo Hospital, a national referral hospital in Jakarta, Indonesia, from August through December 2020.

### Participants

Subjects included CKD patients on haemodialysis who met the inclusion criteria and were recruited using a consecutive sampling method.

The inclusion criteria were as follows: 1) patients over 18 years old who underwent standard haemodialysis treatment twice a week for five hours for at least three months and 2) patients with gastrointestinal complaints (i.e., difficulty defecating, faeces with hard consistency, or a bowel movement frequency of fewer than three times a week). Patients with a history of malignancy, chemotherapy or radiotherapy, patients with autoimmune disorders or receiving immunosuppressants, patients who underwent gut resection, patients with Crohn’s disease or ulcerative colitis, patients whose haemodialysis schedule was altered, patients consuming prebiotics/probiotics/synbiotics, and patients suffering from infection or consuming antibiotics were excluded from this study.

### Intervention

All included subjects underwent history taking, physical examination, and laboratory examination (blood) and provided consent to participate in the study. The subjects then underwent history taking for demographic data, comorbidities and medications as well as physical examination for vital signs, body weight, and body height. We tested the blood sample to evaluate haemoglobin, leucocytes, thrombocytes, urea, creatinine, and albumin to determine baseline characteristics.

After randomization, each subject received 60 capsules containing synbiotics (*Lactobacillus acidophilus* and *Bifidobacterium longum* 5x10^9^ CFU and 60 mg of fructooligosaccharides (FOS)) or 60 capsules containing placebo (Saccharum lactis). The daily dosage was two capsules per day taken every morning before a meal. All subjects were given a compliance card that had to be completed every day after they took the drug. Medication adherence was assessed every 30 days, and the subjects returned any medicine left and showed the compliance card at the subsequent follow-up.

Food intake was assessed before and after the intervention was administered using 24-hour food recall and was carried out by a nutritionist. To evaluate caloric intake and carbohydrates, protein, fat, and fibres consumed by the patient throughout the study, a programme (nutriSurvey) was used. In addition, all participants were asked to continue their dietary habits and previous lifestyle habits and were prohibited from consuming any motility agents during the study period.

After 30 days, a follow-up was performed to evaluate side effects and compliance with the drug regimen. Each subject was then provided with another 60 synbiotic capsules or 60 placebo capsules based on their previous grouping.

The exclusion criteria included subjects who withdrew from the study, those who missed their dose of synbiotics or placebo for more than three consecutive days, those with infections that required antibiotics, those whose haemodialysis schedule changed from twice a week to three times a week or whose treatment modality changed from dialysis to peritoneal dialysis or to renal transplant and those who experienced gastrointestinal symptoms, such as diarrhoea or profuse vomiting requiring hospital admission. Patient adherence to the medication under investigation was expected to reach over 90%.

### Outcome

The primary outcome of this study was a decrease in IS levels. The examination was conducted twice, before and after the intervention was administered. Blood samples were taken predialysis and after each subject fasted for ±8-10 hours. Approximately 100 μL of each subject's serum was added to 900 L of acetonitrile to precipitate the protein. The supernatant was then added to 500 μL of 5 M NaCl for salting-out-assisted liquid–liquid extraction (SALLE) and centrifuged at 14000 rpm for 10 minutes. As a result, two phases were formed: the organic phase (IS in acetonitrile and internal standard) and the aqueous, NaCl and matrix constituent phases. The organic phase was then separated, and as much as 0.2 L was injected into the HPLC system with a fluorescence detector (Agilent Technologies with MassHunterChemStation Software version B04.03.E). IS was measured quantitatively using seven calibration levels with a calibration range of 0.02-100 mg/L. The concentration of IS is expressed in units of mg/L.

The secondary outcome of this study was improvement in constipation-related symptoms and quality of life. They were assessed using the Indonesian-validated Patient Assessment of Constipation: Symptoms (PAC-SYM) and Patient Assessment of Constipation Quality of Life (PAC-QOL) questionnaires [12, 13]. The examination was conducted twice, at the beginning and the end of the study. The PAC-SYM questionnaire includes a 0-4 scale and consists of three parts: abdominal symptoms (questions 1-4), rectal symptoms (questions 5-7), and stool symptoms (questions 8-12). *Symptom improvement* was defined as a decrease in the total PAC-SYM score > 1. The PAC-QOL questionnaire includes a 0-4 scale and consists of four parts: physical discomfort (questions 1-4), psychosocial discomfort (questions 5-12), worries/concern (questions 13-23), and satisfaction (question 24-28). Quality of life improvement was defined as a decrease in the total PAC-QOL score > 1.

### Sample size

To estimate the sample size, a formula for comparing two means was used. With a(n) alpha of 0.05, power of 0.80, and dropout of 20%, the minimum sample size for each group was 30.

### Randomization

We only enrolled subjects who gave consent. A third party (pharmacist) randomized the subjects into two study groups using a computer randomizer. Synbiotics and placebo were both packed on an identical clear gastroenteric coated capsule. Each subject was given one plastic pot containing either intervention or placebo drugs; each identical pot contained 60 capsules. A white paper with information on drug use and administration was placed on the cover of the pot. The drugs were distributed by a third party. None of the patients, researchers, or physicians in charge were aware of the treatment groups.

### Statistical methods

We performed the statistical analysis using SPSS version 20.0. Mean and standard deviation (SD) analyses were performed for numeric data with normal distributions, whereas medians and interquartile ranges (IQRs) were calculated for data with nonnormal distributions. This study utilized intention-to-treat analysis. Bivariate analysis using an independent t test was performed to analyse data with a normal distribution, and the Mann–Whitney U test was performed to analyse data with a nonnormal distribution. A p value of <0.05 was considered statistically significant.

## Results

**Fig. 1:**
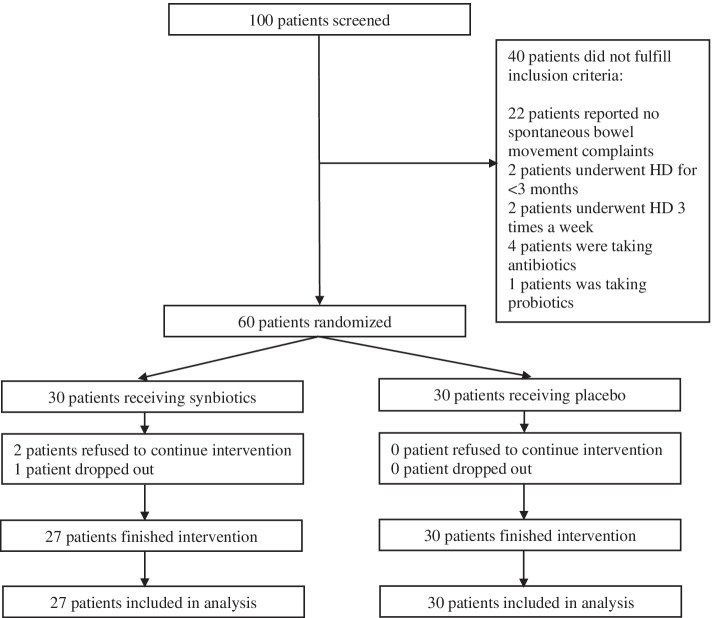
Research algorithm according to Consolidated Standards of Reporting Trials (CONSORT)

Out of 100 screened patients, 60 met the inclusion criteria. These patients were randomized into either the synbiotics or the placebo group. Twenty-seven subjects in the synbiotics group and 30 subjects from the placebo group completed the study. In the synbiotics group, two subjects refused to continue the study, whereas one dropped out due to receiving antibiotics for 15 days during the course of the study. The research algorithm is shown in Fig. 1. Baseline demographic characteristics as well as IS levels and PAC-SYM and PAC-QOL scores are provided in Table 1.

**Table 1:** Initial characteristics of study subjects

Characteristics	Synbiotics (*N*=30)	Placebo (*N*=30)	*p* value
Gender, n(%)			
Male	10 (33.3)	11 (36.7)	0.787^a^
Female	20 (66.7)	19 (63.3)	
Age (years), mean (SD)	51.23 (13.57)	52.33 (11.29)	0.734^b^
HD duration (months), median (IQR)	70.50 (46.25-118.75)	57.50 (29.5-99.7)	0.329^c^
Comorbidities, n(%)			
- Hypertension	25 (83.3)	25 (83.3)	0.635^d^
- Diabetes	11 (36.7)	8 (26.7)	0.405^a^
- Heart failure	5 (16.7)	5 (16.7)	0.635^d^
- Coronary heart disease	4 (14.3)	2 (6.7)	0.335^d^
- Stroke	1 (3.3)	3 (10)	0.306^d^
Medication taken, n(%)			
- ACEi/ARB	12 (40.0)	14 (46.7)	0.602^a^
- CCB	20 (66.7)	18 (60.0)	0.592^a^
- Beta blocker	3 (10.0)	9 (30)	0.053^a^
- Alpha blocker	14 (46.7)	9 (30)	0.184^a^
- Insulin	8 (26.7)	3 (10)	0.095^a^
- Oral hypoglycaemic drugs	3 (10.0)	4 (13.3)	0.500^d^
- Phosphate binder	28 (93.3)	27 (90.0)	0.640^d^
- Oral iron supplements	1 (3.3)	3 (10.0)	0.306^d^
HD adequacy (Kt/V), mean (SD)	1.92 (0.35)	1.83 (0.30)	0.284^b^
Body mass index (kg/m^2^), mean (SD)	22.56 (4.80)	24.22 (4.64)	0.180^b^
Laboratory			
- Haemoglobin (g/dL), median (IQR)	9.4 (7.9-10.9)	9.4 (8.80-10.42)	0.487^c^
- Leucocyte (/uL), mean (SD)	8142 (2380.9)	7978 (1783.9)	0.764^b^
- Thrombocyte (/uL), mean (SD)	257933.33 (61952.11)	244433.33 (63318.39)	0.407^b^
- Albumin (mg/dL), mean (SD)	3.90 (0.28)	3.87 (0.4)	0.789^b^
- Urea (mg/dL), mean (SD)	144.77 (44.83)	131.94 (38.45)	0.148^b^
- Creatinine (mg/dL), mean (SD)	11.86 (3.46)	12.35 (3.7)	0.547^b^
Dietary intake			
- Calories (kcal/kg/day), median (IQR)	28.82 (22.32-36.34)	26.84 (18.41-36.21)	0.220^c^
- Protein (gram/kg/day), mean (SD)	0.91 (0.38)	0.79 (0.30)	0.178^b^
- Carbohydrate (gram/day), mean (SD)	183.98 (71.05)	206.99 (94.99)	0.293^b^
- Fat (gram/day), mean (SD)	70.02 (20.9)	64.49 (20.51)	0.306^b^
- Fibre (gram/day), mean (SD)	6.09 (3.76)	6.11 (3.33)	0.980^b^
Indoxyl sulphate (mg/L), median (IQR)	26.98 (22.78-34.77)	20.95 (17.25-27.07)	0.062^c^
PAC-SYM score, median (IQR)	8 (5-11.25)	6 (4-9.5)	0.137^c^
PAC-QOL score, median (IQR)	17.5 (14-24)	18 (13-26.25)	0.970^c^

*ACEi *angiotensin-converting enzyme inhibitor*, ARB *angiotensin receptor blocker*, CCB *calcium channel blocker*, HD *haemodialysis*, PAC-SYM *Patient Assessment of Constipation Symptoms*, PAC-QOL *Patient Assessment of Constipation Quality of Life

Data were analysed using the ^a^chi-square test, ^b^t test, ^c^Mann–Whitney U test, and ^d^Fisher test

### Effects of synbiotic supplementation on indoxyl sulphate toxin levels

In the synbiotics group, the median IS toxin levels pre- and postintervention were 26.98 mg/L (22.78-34.77 mg/L) and 27.94 mg/L (23.25-34.05 mg/L), respectively. The initial and posttreatment median IS toxin levels of the placebo group were 20.95 mg/L (17.25-27.07 mg/L) and 22.88 mg/L (18.20-29.38 mg/L), respectively (Table 2). The synbiotics group showed an increased level of IS of 0.17 mg/L (-2.67-4.89 mg/L), whereas the placebo group showed an increased level of 0.67 mg/L (-3.01-2.14 mg/L). A nonparametric Mann–Whitney bivariate analysis test was used to compare the difference in IS levels pre- and postintervention in both groups. In Table 2, compared to placebo, no significant changes in IS levels were observed after synbiotic administration (p=0.438).

**Table 2:** The effects of synbiotics and placebo supplementation on indoxyl sulphate levels

Indoxyl sulphate	SYNBIOTICS (*N*=30)				PLACEBO (*N*=30)				P
	Pre	Post	Δ	P	Pre	Post	Δ	P	
Median (IQR) (mg/dL)	26.98 (22.78-34.77)	27.94 (23.25-34.05)	-0.17 (-2.67-4.89)	0.728^a^	20.95 (17.25-27.07)	22.88 (18.20-29.38)	-0.67 (-3.01-2.4)	0.586 ^a^	0,438^b^

^a^Pre- and postintervention were analysed using the Wilcoxon test

^b^DeltaIS between groups was analysed using the Mann–Whitney test

### The effect of synbiotic administration on constipation symptoms according to the PAC-SYM questionnaire

Table 3 shows a significant difference in abdominal symptoms (questions 1-4) between the synbiotics and placebo groups (p = 0.023). We also observed significant improvement in rectal symptoms (questions 5-7) and stool symptoms (questions 8-12) after administering synbiotics. There was an overall improvement in constipation in the group that received synbiotics compared to the placebo group (*p* = 0.006). This study also analysed dietary intake before and after intervention and found no difference in dietary pattern in terms of caloric intake and protein, carbohydrate, fat, and fibre consumption, which can affect defecation patterns.

**Table 3:** The effects of synbiotics and placebo supplementation on constipation symptoms based on PAC-SYM questionnaire

PAC-SYM	SYNBIOTICS (*n*=30)		PLACEBO (*n*=30)		P
	Pre	Post	Pre	Post	
Abdominal symptoms (questions 1-4), median (IQR)	3 (0-9)	0 (0-3)	2 (0-9)	1 (0-8)	0.023^a^
Rectal symptoms (questions 5-7), median (IQR)	0 (0-7)	0 (0-2)	0.5 (0-7)	0 (0-4)	0.025^a^
Stool symptoms (questions 8-12), median (IQR)	4 (0-14)	1 (0-6)	3 (0-12)	2 (0-7)	0.047^a^
Total score, median (IQR)	8 (2-25)	1 (0-8)	6 (1-28)	5.5 (0-17)	0.006^a^

^a^Postintervention data were analysed using the Mann–Whitney test

Table 4 shows significant improvement in physical discomfort (questions 1-4), psychosocial discomfort (questions 5-12), and worries/concerns (questions 13-23) after synbiotic administration compared to placebo. However, synbiotic administration did not show significant results in terms of satisfaction (questions 24-28). Synbiotic administration showed an overall significant difference in quality of life caused by constipation (*p*=0.001).

**Table 4:** The effects of synbiotics and placebo supplementation on constipation-related quality of life based on PAC-QOL questionnaire

PAC-QOL	SYNBIOTICS (*n*=30)		PLACEBO (*n*=30)		P
	Pre	Post	Pre	Post	
*Physical discomfort* (questions 1-4), median (IQR)	2 (0-8)	0 (0-2)	2 (0-11)	0.5 (0-10)	0.007^a^
*Psychosocial discomfort* (questions 5-12), median (IQR)	2 (0-16)	0 (0-3)	3 (0-12)	2 (0-11)	0.000^a^
*Worries/concerns* (questions 13-23), median (IQR)	6 (2-29)	0 (0-9)	4.5 (0-24)	3 (0-12)	0.001^a^
*Satisfaction* (questions 24-28), median (IQR)	8 (4-12)	9 (7-13)	9.5 (2-17)	8.5 (4-14)	0.609^a^
Total score, median (IQR)	18 (10-57)	11 (8-21)	18 (10-44)	16 (7-33)	0.001^a^

^a^Postintervention data were analysed using the Mann–Whitney test

**Table 5:** Postintervention adverse events

Components	Synbiotics (*n*=30) N (%)	Placebo (*n*=30) N (%)	*p* value
Adverse events (n,%)			
- Diarrhoea	6 (20)	2 (6.7)	0.259^a^
- Nausea/vomiting	2 (6.7)	3 (10)	
- Bloating	1 (3.3)	0 (0)	
- Stomachache/heartburn	0 (0)	2 (6.7)	
Hospitalization	0 (0)	0 (0)	-
Death	0 (0)	0 (0)	-

^a^ Data were analysed using the chi-square test

### Adverse events observed during the course of the study

During the course of this study, six subjects in the synbiotics group and two subjects in the placebo group complained of diarrhoea (two to five times a day) during the first two weeks of intervention. Five subjects showed improvement without antidiarrheal treatment, and three subjects required antidiarrheal treatment to recover. Despite recovery in all patients suffering from diarrhoea, two subjects in the synbiotics group opted to discontinue their participation in this study and were therefore considered dropouts. Two subjects in the synbiotics group and three subjects in the placebo group experienced nausea. One subject in the placebo group needed a proton pump inhibitor for two days, whereas the rest improved without additional treatment. As shown in Table 5, one subject in the synbiotics group felt bloated, and two subjects in the placebo group complained of colicky pain, but all improved without any additional treatment. No subjects died or required hospitalization.

Six subjects (out of 27) in the synbiotics group and seven subjects (out of 30) in the placebo group had an adherence of less than 100%. The mean adherence in subjects who completed this study was 99.4% and 99.2% in the synbiotics and placebo groups, respectively.

## Discussion/conclusion

The excretion of uraemic toxins, such as IS, decreases with declining kidney function. Although haemodialysis is an advanced kidney replacement therapy, IS still cannot be completely eliminated. This is because this toxin is large, and 90% is bound to albumin [14]. Various strategies to improve IS removal, including (using) haemodiafiltration machines, increasing dialysate flow, increasing dialyzer membrane size, or adding a sorbent to the dialysate, have not shown promising results [15–17]. This suggests the need for an alternative way to lower the level of IS in CKD or CKD-HD patients. It was expected that the administration of synbiotics would suppress IS production in the gastrointestinal tract by improving gut microbiota dysbiosis. A previous study by Cossola C et al. found that a combination of prebiotics and probiotics restored the balance of gut microbiota and suppressed IS production [18]. However, some studies and a recent meta-analysis showed no correlation between synbiotics and IS reduction in CKD-HD subjects [8, 9, 19, 20]. Likewise, our study found nonsignificant results.

The reasons behind these findings might be because a 60-day administration and observation period were not sufficient to demonstrate a significant IS reduction. Thus, a longer observation period and serial examination of IS might be needed. Another factor that might have played a role in the findings is the fact that a previous study used a synbiotic combination that differed from ours. The synbiotics used by previous studies contained different strains of bacteria and higher amounts of prebiotics, which might have led to various effects [ 8,9,18–20]. Furthermore, the results of these studies show the importance of determining the type, dosage and duration of administration of synbiotics to suppress IS levels [8, 9, 18–20]. Additionally, this illustrates the need for faecal microbiota analysis to determine the most suitable synbiotics to be given to CKD-HD patients. In Indonesia, intestinal microbiota profile examination has never been performed on CKD-HD patients, and to date, there are no accurate data on the most suitable synbiotics for CKD-HD patients in Indonesia.

Saccharolytic bacteria provide benefits in improving intestinal microbiota homeostasis, lowering the amount of pathogenic bacteria, improving intestinal transit time, increasing the frequency of defecation, improving faecal consistency, decreasing bloating symptoms and producing butyric acid to increase intestinal barrier functions [21]. The presence of saccharolytic bacteria is expected to lower the amount and activity of proteolytic bacteria, which lowers the level of uraemic toxins, such as IS. The presence of fructooligosaccharides (FOS) modulates the growth of bacteria, such as *Bifidobacterium,* and increases the ratio of *Roseburia*/*E. rectal*, which plays a role in increasing the level of butyrate acid, which in turn serves as the source of energy in the regeneration of cells in the host’s intestines. Fructooligosaccharides also serve as substrates for saccharolytic bacteria contained in the synbiotic preparation, leading to increased SCFA production and suppressed uraemic toxin production by proteolytic bacteria [22].

Patients with CKD experience constipation more often than the normal population. This is due to the restriction of fibre and water, reduced physical activity, use of medications such as oral iron supplements, and gut microbiota dysbiosis in haemodialysis patients [23]. An in vitro study reported an inflammation process due to gut-derived uraemic toxin disrupting intestinal motility. Nishiyama *et al*. demonstrated that rats with CKD also had gut dysbiosis, reduced intestinal motility, reduced amounts of faeces, and intestinal inflammation [24]. A study by Ramos *et al*. showed that CKD patients suffering from constipation tended to have higher uraemic toxin levels [25]. Constipation increases colonic transit time, leading to an increase in the activity of proteolytic bacteria in metabolizing amino acids and producing uraemic toxins. The presence of uraemic toxin itself may worsen constipation. In other words, constipation and gut dysbiosis affect each other [26]. A study by *Salmean et al*. showed an improvement in defecation frequency among 13 CKD patients receiving high fibre supplementation for 12 weeks [27]. In our study, we found that the administration of synbiotics capsules improved complaints related to constipation symptoms patterns, as shown in the improved score on the PAC-SYM questionnaire. This is because the administration of synbiotics increases the amount and activity of saccharolytic bacteria in producing butyric acid. Butyric acid is the source of energy for the regeneration of colonocytes; thus, the increase in its production improves intestinal motility and contractility, lowering colonic transit time. Additionally, improving gut dysbiosis decreases intestinal inflammation, which causes disturbances in intestinal motility [6, 28].

Constipation, if not well treated, may affect the quality of life in haemodialysis patients. Zhang *et al*. reported that haemodialysis patients with constipation symptoms tended to have poorer quality of life and were prone to depression compared to subjects without constipation [7]. Ranganathan *et al*. reported that the administration of probiotics for six months in stage 3-4 CKD patients increased their quality of life [29]. A study by *Haghihat et al*. showed that the administration of synbiotics in haemodialysis patients improved their mental status, including depression and anxiety symptoms, but was not associated with significant improvement in terms of quality of life [30]. The results of this study were consistent with those of previous studies, which showed an improvement in terms of constipation-related quality of life as assessed using the PAC-QOL questionnaire. We observed significant improvements in terms of physical discomfort, psychosocial discomfort, and worries, although we did not observe significant improvement in terms of patient satisfaction. To date, current studies have shown various results. The pathogenesis of how synbiotics may affect the quality of life and the possibility of a gut-brain axis role are not yet completely understood. Hence, the need for further studies remains.

To the best of our knowledge, this is the first study in Indonesia that successfully showed the efficacy of synbiotic supplementation on the improvement of constipation symptoms and constipation-related quality of life in patients undergoing haemodialysis, although its efficacy in decreasing the IS level has not yet been established. This study has several limitations. We did not perform genomic analysis of gut microbiota on patients’ faecal samples to assess dysbiosis patterns in Indonesian CKD-HD patients and determine whether there was any change in dysbiosis patterns after synbiotic administration. Moreover, this study did not measure other uraemic toxins, such as p-cresyl sulphate and indole acetic acid, which may also increase gut microbiota dysbiosis. In addition, this study only included subjects who received haemodialysis twice a week, so attention is needed to generalize this study's results.

In conclusion, the administration of synbiotics containing *Bifidobacterium longum*, *Lactobacillus acidophilus* (5x10^9^ CFU), and 60 grams of *fructo-oligosaccharides (*FOS) in two capsules per day for 60 days has not been shown to reduce levels of IS toxin but can improve constipation symptoms and quality of life associated with constipation in CKD-HD patients.

## Supplementary Information

**Additional file 1.**

## Data Availability

The data that support the findings of this study are available from the corresponding author, AL, upon reasonable request.

## Abbreviations

CKD-HD: Chronic Kidney Disease on Haemodialysis
CFU: Colony-Forming Unit
FOS: Fructoooligosaccharides
IS: Indoxyl Sulphate
P-CS: P-Cresyl Sulphate
IAA: Indole Acetic Acid
SCFA: Short Chain Fatty Acid
NF-Ƙb: Nuclear Factor-Ƙb
PAI: Plasminogen Activator Inhibitor
PAC-SYM: Patient Assessment of Constipation-Symptoms
PAC-QOL: Patient Assessment of Constipation Quality of Life
SD: Standard Deviation
IQR: Interquartile Range
SCFA: Short-Chain Fatty Acids
