# Supplementary material for: The effects of synbiotics on indoxyl sulphate level, constipation, and quality of life associated with constipation in chronic haemodialysis patients: a randomized controlled trial
Source: BMC Nephrol. 2022 Jul 22;23:259. doi: 10.1186/s12882-022-02890-9 (PMC9308250; doi:10.1186/s12882-022-02890-9)
Supplement: Supplementary file 1 — Additional file 1. [file 12882_2022_2890_MOESM1_ESM.docx]

Supplementary table 1. Daily Food Recall Analysis

| Intake | Synbiotics | | p | Placebo | | p |
| --- | --- | --- | --- | --- | --- | --- |
|  | Pre | Post |  | Pre | Post |  |
| Calories (kcal/kg/day), median IQR | 28.82 (22.32-36.34) | 28.14 (22.51-42.41) | 0.719 | 26.84 (18.41-36.21) | 24.58 (18.58-35.27) | 0.926 |
| Protein (gram/kg/day), mean(SD) | 0.91 (0.38) | 0.89 (0.42) | 0.412 | 0.79 (0.30) | 0.87 ( 0,41) | 0.203 |
| Carbohydrate (gram/day), mean(SD) | 183.98 (71.05) | 202 (70.2) | 0.245 | 206.99 (94.99) | 191,39 (97,6) | 0.259 |
| Fat (gram/day),mean (SD) | 70.02 (20.09) | 67.15 (24.32) | 0.52 | 64.49 (20.51) | 71.29(23.56) | 0.122 |
| Fiber (gram/day), mean(SD) | 6.09 (3.76) | 7.53 (3.54) | 0.235 | 6.11 (3.33) | 7.31(3.93) | 0.09 |

SD: Standard deviation, IQR: interquartile range
